# Influence of Age on Brain Edema Formation, Secondary Brain Damage and Inflammatory Response after Brain Trauma in Mice

**DOI:** 10.1371/journal.pone.0043829

**Published:** 2012-08-30

**Authors:** Ralph Timaru-Kast, Clara Luh, Philipp Gotthardt, Changsheng Huang, Michael K. Schäfer, Kristin Engelhard, Serge C. Thal

**Affiliations:** 1 Department of Anesthesiology, University Medical Center, Johannes Gutenberg-University, Mainz, Germany; 2 Focus Program Translational Neuroscience, Johannes Gutenberg-University, Mainz, Germany; Julius-Maximilians-Universität Würzburg, Germany

## Abstract

After traumatic brain injury (TBI) elderly patients suffer from higher mortality rate and worse functional outcome compared to young patients. However, experimental TBI research is primarily performed in young animals. Aim of the present study was to clarify whether age affects functional outcome, neuroinflammation and secondary brain damage after brain trauma in mice. Young (2 months) and old (21 months) male C57Bl6N mice were anesthetized and subjected to a controlled cortical impact injury (CCI) on the right parietal cortex. Animals of both ages were randomly assigned to 15 min, 24 h, and 72 h survival. At the end of the observation periods, contusion volume, brain water content, neurologic function, cerebral and systemic inflammation (CD3+ T cell migration, inflammatory cytokine expression in brain and lung, blood differential cell count) were determined. Old animals showed worse neurological function 72 h after CCI and a high mortality rate (19.2%) compared to young (0%). This did not correlate with histopathological damage, as contusion volumes were equal in both age groups. Although a more pronounced brain edema formation was detected in old mice 24 hours after TBI, lack of correlation between brain water content and neurological deficit indicated that brain edema formation is not solely responsible for age-dependent differences in neurological outcome. Brains of old naïve mice were about 8% smaller compared to young naïve brains, suggesting age-related brain atrophy with possible decline in plasticity. Onset of cerebral inflammation started earlier and primarily ipsilateral to damage in old mice, whereas in young mice inflammation was delayed and present in both hemispheres with a characteristic T cell migration pattern. Pulmonary interleukin 1β expression was up-regulated after cerebral injury only in young, not aged mice. The results therefore indicate that old animals are prone to functional deficits and strong ipsilateral cerebral inflammation without major differences in morphological brain damage compared to young.

## Introduction

Traumatic brain injury (TBI) is a serious public healthcare burden and the most common cause for trauma related death and disability in industrialized countries affecting over 55 million people worldwide [1], [2]. The majority of traumatic deaths in young and aged patients are caused by severe brain injury. There is a bimodal distribution of TBI with respect to age. Incidence peaks between the ages 15 and 24 and after 75 years of age. Starting with 65, the incidence rate of TBI doubles every additional ten years of age. Beyond the age of 75 the highest TBI-related hospitalization rate was observed [3]. Elderly individuals with age of 65 or older account for a disproportionate high number of TBI cases and have a higher chance for poor outcome [4]. Patients older than 65 years who survived a mild TBI have a poorer functional outcome at 6 months compared to younger patients [5]. Although advanced age is associated with poor outcome following TBI and age has been shown to be a primary determinant of survival following isolated TBI [6]–[8], experimental research is primarily performed in young animals [9], [10]. In young animals the pathophysiologic cascade after impact (primary injury) is well characterized and multiple studies aim to limit secondary brain damage in the pericontusional tissue. The mechanisms behind the poor outcome of aged versus young individuals have been addressed only in a few studies [9]. So far it is still unclear whether the difference in outcome is a result of a more severe secondary brain damage [11], increased neuroinflammation worsening secondary brain damage [12], or reduced capability of old individuals to compensate neurological deficits [13].

The aim of the present study was to clarify whether age affects functional outcome, systemic and cerebral inflammation as well as secondary brain damage in young (2 months) and aged (21 months) mice subjected to a controlled cortical impact injury (CCI).

## Results

### Mortality

#### Mortality during housing of animals

**Figure 1:**
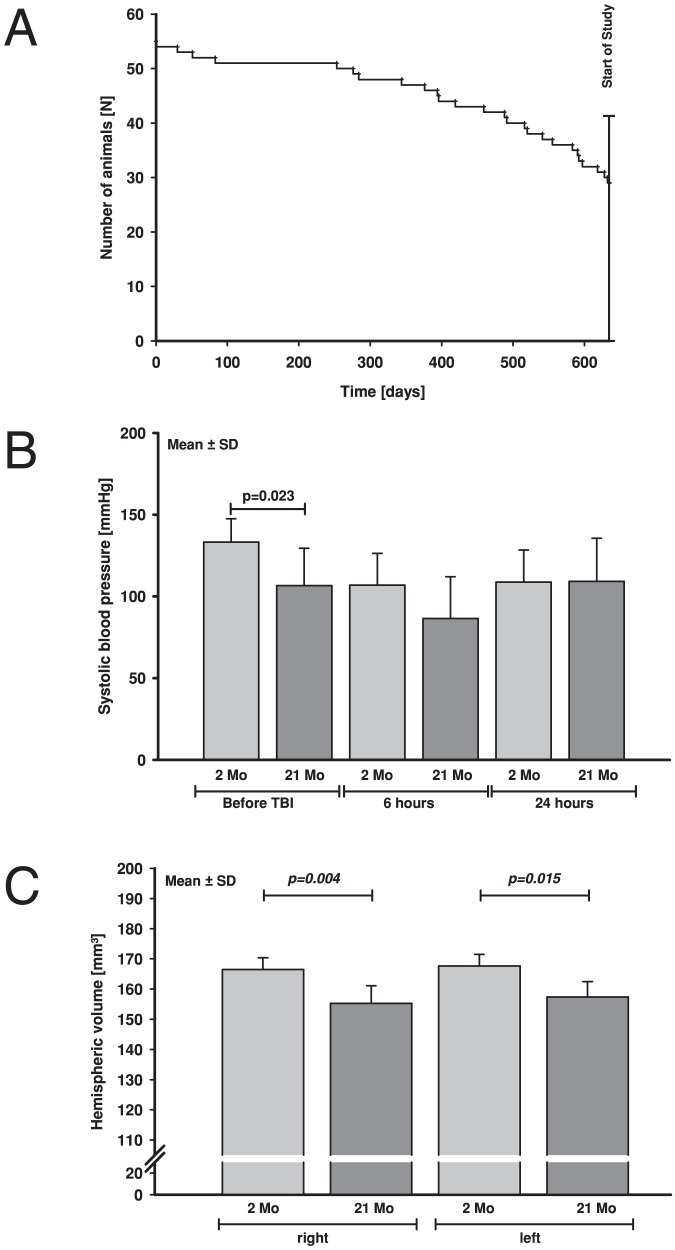
Characteristics of the old animals used in the present study. **A:** Kaplan-Meyer curve of the survival rate of the old animal group. Animals lived for 630 day in the animal facility until the start of the experiments. Out of 55 animals, 29 mice survived this time span (survival rate 45%). The survival curve demonstrates a dramatic increase of mortality during the last months before the experiments. **B:** Systolic blood pressure [mmHg] measured non-invasively in awake animals of both age groups before CCI and 6 hours and 24 hours after experimental TBI. Before trauma systolic blood pressure of young animals was higher than in aged mice. **C:** Histological evaluation of brain hemisphere volumes of young (light grey, n = 6) and old (dark grey n = 6) naïve animals revealed that both hemispheres were significantly larger in the young (p<0.05) naïve mice (2 months).

Animals stayed for 630 days in our animal facility until the start of the experiments. Out of 55 animals, 29 mice survived this time span (cumulative mortality 45%, see Kaplan Meyer curve, **Figure 1 A**).

#### Experimental mortality

All young animals survived the observation period. In contrast, old animals showed a high overall mortality rate of 19.2% (5/26). In the 24-hours survival group 3 of 17 (17.6%) and in the 72-hours survival group 2 of 9 (22%) old animals died before end of the experiments.

### Physiological parameters

Physiological parameters (pH, arterial pCO_2_ and pO_2_) determined 15 minutes after CCI were within normal range in both age groups. Body temperature and systolic blood pressure during surgery were not different between groups and were within physiological levels (**Table 1** **and** **2**). Systolic blood pressure was measured before CCI as well as 6 and 24 hours after CCI. Before CCI old animals had lower blood pressures compared to young animals (21 months: 107±23 vs. 2 months: 133±14 mmHg; p<0.05, **Figure 1 B**). After CCI no differences were detectible between age groups. At 6 hours after CCI blood pressure decreased in old and young mice (21 months: 87±26 mmHg; 2 months: 107±19 mmHg) and recovered after 24 hours (21 months: 110±26 mmHg; 2 months: 109±20 mmHg).

**Table 1:** Physiological Parameters I: peritraumatic blood pressure and body temperature.

groups	15 min survival		24 h survival		72 h survival	
	2 months	21 months	2 months	21 months	2 months	21 months
**Systolic blood**	99±7	97±8	100±13	80±14	97±17	86±26
**pressure before**						
**CCI [mmHg]**						
**Systolic blood**	96±7	96±11	111±15	88±20	100±13	93±28
**pressure after**						
**CCI [mmHg]**						
**Body tempera-**	36.7±0.4	36.7±0.3	37.7±0.6	36.7±0.3	37.2±0.6	36.5±0.7
**ture before CCI**						
**[°C]**						
**Body temper-**	37.0±0.3	37.2±0.7	37.6±0.4	36.9±0.4	37.3±0.4	36.8±0.5
**ture after CCI**						
**[°C]**						

Physiological Parameters are presented as mean ± SD and were not significantly different between age groups.

**Table 2:** Physiological Parameters II (blood gas analysis).

Group	2 months (15 min survival)	21 months (15 min survival)
**pH**	7.32±0.04	7.31±0.04
**paCO_2_ [mmHg]**	48±4	48±5
**paO_2_ [mmHg]**	272±28	247±21
**Hb [g/dL]**	13.9±0.3 *	12.1±0.9 *
**Glucose [mg/dL]**	195±118	150±49

Perioperative arterial blood pH, paCO_2_, paO_2_, and glucose concentration were not statistically significant different between both age groups. Hemoglobin concentration (Hb) were significantly lower in aged animals (*p<0.05, mean±SD).

### Hemispheric brain volume

Brain sizes of naïve animals were compared between both age groups. Brains of 2 months old animals were 8% larger compared to 21 months old animals (p<0.05; **Figure 1 C**).

### Histological damage

**Figure 2:**
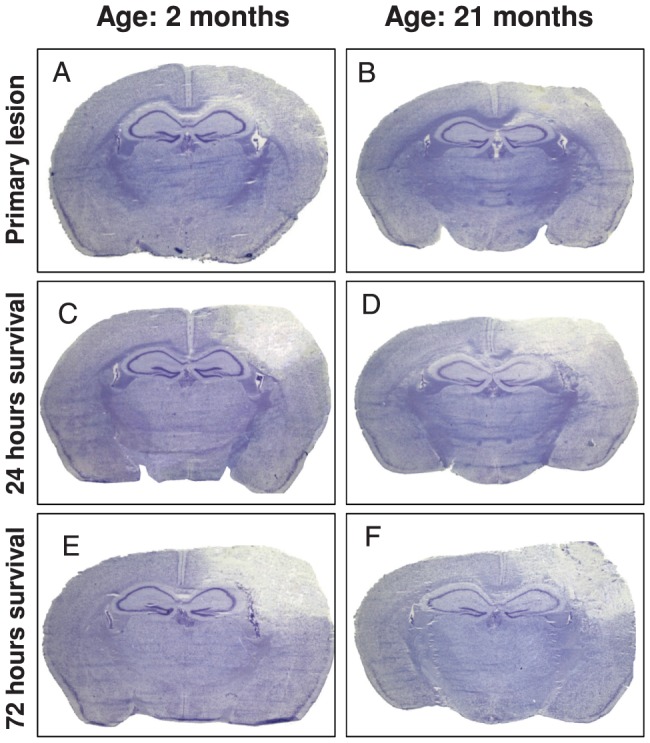
Typical pictures of Nissl-stained coronal sections at bregma level −0.98 to −1.28 mm of 2 months and 21 months old mice at 15 minutes (A; B), 24 (C; D) and 72 (E; F) hours after controlled cortical impact injury. The pictures demonstrate the lesion growth within the first three days after traumatic brain injury without difference between age groups (see also quantification of the lesion, **Figure 3 A, B**).

**Figure 3:**
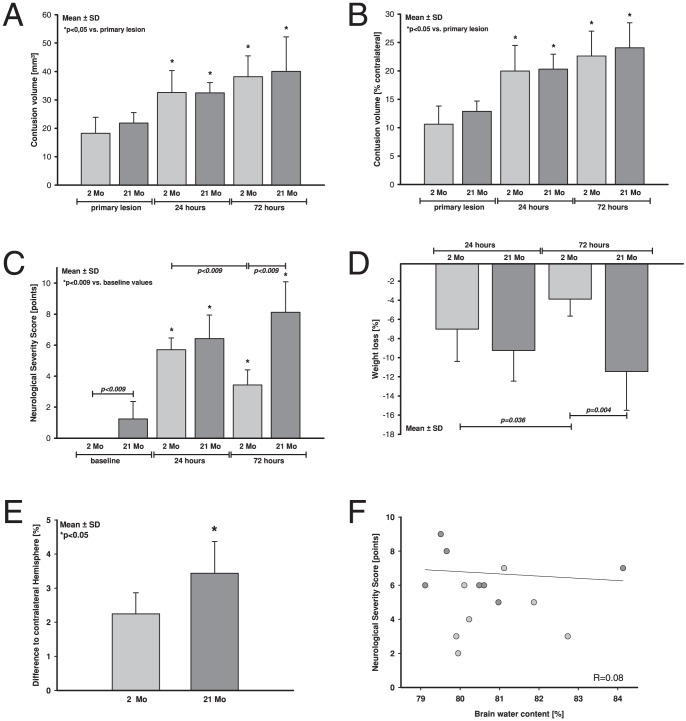
Contusion Volume, neurological function, and brain edema formation after TBI in young and old mice. **A and B:** Contusion volumes were assessed in Nissl-stained brain sections in young (2 months, light grey) and old (21 months, dark grey) C57/Bl6 mice at 15 minutes ( = 15 m, n = 6), 24 hours ( = 24 h, n = 7) and 72 hours ( = 72 h, n = 7) after CCI and is presented as absolute values (**A**; mm^3^) and in percentage of the contralateral hemisphere (**B**; %). 15 min-groups represent the primary lesion group. 24 hours after CCI contusion volume increased significantly in both groups. After 72 hours there was an additional increase of brain damage volume in both groups without difference between age groups. **C:** To determine the neurological function in these animals a Neurological Severity Score (NSS) was performed before CCI (baseline values; additionally data of naïves included), and 24 hours and 72 hours after CCI. 24 hours after CCI NSS increased in both groups without significant difference between young and old animals. 72 hours after TBI neurological impairment improved in young animals, while motor function deteriorated in old animals. **D:** Body weight loss (in percent of initial weight) was determined as an additional marker for the well being of the animals and showed similar results to the neurological data. After 24 hours weight loss was present in both age groups. However, 72 hours after insult young, but not old animals started to regain weight. **E:** Brain water content was assessed 24 hours after CCI in young (2 months, light grey, n = 7) and old (21 months, dark grey, n = 7) and is presented as difference to the contralateral hemisphere. Increase in brain water content was significantly higher in aged animals, but (**F**) nonlinear regression analysis failed to show a correlation between neurological function and brain water content (r = 0.08).

Brain contusion volume was determined in Nissl stained sections (**Figure 2** **,** **3 A**) and increased significantly in both age groups from 18.2±5.6 and 21.9±3.7 mm^3^ (primary injury, 15 min after TBI) to 32.6±7.7 and 32.5±3.6 mm^3^ after 24 hours, and 38.2±7.3 and 40.1±12.1 mm^3^ after 72 hours in young and old animals, respectively. To correct for differences in brain size, lesion volumes were also calculated as percentage of the contralateral hemispheres (**Figure 3 B**). Lesion volume increased significantly 24 hours after CCI compared to primary injury (2 months: 10.6±3.2%; 21 months: 12.9±1.8%) to 20.0±4.5% (2 months) and 20.3±2.6% (21 months; p<0.05). After 72 hours there was an additional expansion of lesion volume in both groups (2 months: 22.6±4.4%: 21 months: 24.0±4.4%). However, brain lesion was not statistically different at any time point between age-matched groups.

### Neurofunctional outcome

Neurological function was determined with a Neurological Severity Score (NSS, [14]) one hour before CCI (baseline), and 24 and 72 hours after CCI (**Figure 3 C**). Before CCI all young animals performed the tasks without neurological deficit (0±0 points); whereas some old animals showed a slightly impaired score (1.2±1.1 points; p<0.009). This was caused by their large body sizes, which prevented some old mice from successfully performing the balance and beam-walking task. Neurological function was significantly impaired in both age groups 24 hours after CCI (p<0.009 vs. baseline) without difference between young and old animals (2 months: 5.7±0.8 points; 21 months: 6.4±1.5 points). 72 hours after TBI neurological impairment improved in young animals, while neurological function of old animals deteriorated compared to 24 hours post injury (2 months: 3.4±1.0 points; 21 months: 8.1±2.0 points; p<0.009, **Figure 3 C**).

As a broad marker for general well-being we determined body weight loss (percentage of initial body weight, **Figure 3 D**). 24 hours after insult weight loss was not different between both groups (2 months: −7.0±3.4%; 21 months: −9.3±3.2%). However, 72 hours after CCI young animals regained body weight (p = 0.036 vs. 24 hours), while old animals continued to lose body weight (2 months: −3.9±1.8%; 21 months: −11.4±4.0%; p = 0.004).

### Brain water content

In a separate set of animals, brain water content was determined 24 hours post insult. Brain water content was not significantly different between age groups (ipsilateral: 81.0±1.1% and 80.9±1.8%, contralateral: 79.0±1.3% and 77.2±1.3% for young and old mice, respectively). However, increase in ipsilateral brain water content, calculated as percent difference between ipsilateral to contralateral values, was higher in aged mice (3.4±0.9%), compared to young animals (2.2±0.6%; **Figure 3 E**, p<0.05).

Regarding neurological outcome, NSS values in old animals were higher (NSS 5.0±1.8) compared to young mice (NSS 3.8±1.2; p<0.05). However a nonlinear regression analysis between NSS values and absolute brain water content revealed no correlation between brain edema and poor neurological outcome (r = 0.08; **Figure 3 F**).

### Cerebral mRNA expression of IL1β, IL6 and TNFα

IL1β, IL6, and TNFα mRNA expression was selected as representative inflammatory cytokines and were quantified at 15 minutes, 24, and 72 hours post injury in injured and contralateral hemispheres. Baseline mRNA expression of IL1β (1.3-fold), TNFα (2.5-fold) and IL6 (2-fold) were already significantly higher in 21 months old animals compared to 2 months (**Table 3**) indicating a presence of a subclinical chronic inflammatory process in the elderly, referred to as “inflamm-aging” [15]. To normalize for differences in naïve brain tissue, the post-traumatic expression data was therefore calculated as % of naïve animals (of each age group).

**Table 3:** Assessment of cerebral inflammatory cytokine expression in naïve animals.

	2 months	21 months	2 months	21 months
	right	right	left	left
**IL1β**	10.0±1.4	13.8±3.6 *	11.6±2.2	13.9±6.0
[mRNA copies *10^−5^]				
**TNFα**	1.0±0.4	2.5±1.2 *	1.1±0.5	2.5±0.8 *
[mRNA copies *10^−5^]				
**IL6**	3.0±0.7	6.5±1.4 *	4.6±1.3	10.3±3.0 *
[mRNA copies *10^−6^]				

IL1β, TNFαand IL6 expression of young (2 months) and old (21 months) were determined in the right and left hemisphere of animals without injury. The expression of these cytokines was significantly higher in old naïve animals, compared to young mice. Absolute copy numbers of the target genes were normalized against the absolute copy numbers of Cyclophilin A (PPIA) as control gene. Data are presented as mean±SD (*p<0.05, 2 months vs. 21 months).

**Figure 4:**
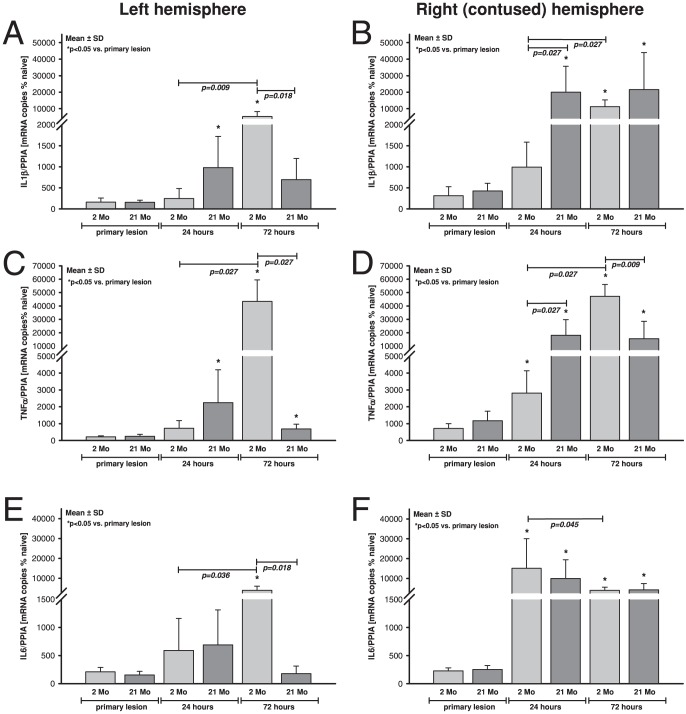
Intracerebral gene expression of inflammatory cytokines in young and old animals after TBI. Differential mRNA expression of interleukin 1β (IL1β; **B**), tumor-necrosis-factor α (TNFα; **C** and **D**) and interleukin 6 (IL6; **E** and **F**) were determined in young (2 months, light grey) and old (21 months, dark grey) C57/Bl6 mice at 15 minutes ( = 15 m, n = 6), 24 hours ( = 24 h, n = 7) and 72 hours ( = 72 h, n = 7) after CCI (data are normalized to the housekeeping gene cyclophilin A and are presented as mean±SD in % of age matched naïve animals; p-values were adjusted for multiple comparison by Bonferroni).

Already 15 minutes after insult IL1β expression (**Figure 4 A, B**) increased ipsilateral to lesion and was independent of age to 315±213% of naïve (2 months) and 427±182% of naïve (21 months), while expression remained stable in the contralateral hemispheres (2 months: 163±98% of naïve; 21 months: 156±52% of naïve). The expression reached a maximum in aged animals after 24 hours (20037±15676% of naïve) compared to young animals (995±590% of naïve). In contrast, young animals demonstrated a delayed increase 72 hours post insult (11229±4112% of naïve), which was also less pronounced compared to old animals (21653±22305% of naïve). In the contralateral hemisphere IL1β expression was not influenced at 24 hours after trauma in 2 months old animals (247±239% of naïve), whereas in old animals IL1β expression increased significantly and reached a maximum of 980±744% of naïve. In contrast, young animals demonstrated a delayed and stronger peak at 72 hours after TBI (5302±2947% of naïve), while IL1β expression dropped in old animals to 697±504% of naïve. The data suggests a late and bilateral onset of IL1β expression in young animals, whereas in old animals IL1β increases primarily ipsilateral to damage within 24 hours.

The gene expression of TNFα profile showed a similar pattern of an early primarily unilateral inflammatory response in old animals vs. a late and global up-regulation in young animals (**Figure 4 C, D**). In young animals TNFα expression increased with a delayed peak 72 hours post insult in the ipsilateral (15 min: 710±285% of naïve; 24 h: 2814±1323% of naïve; 72 h: 47204±8849% of naïve) and contralateral hemispheres (15 min: 215±63% of naïve; 24 h: 721±456% of naïve; 72 h: 43406±16053% of naïve). In aged animals TNFα expression peaked already 24 hours post insult primarily ipsilateral (15 min: 1168±571% of naïve; 24 h: 18041±11765% of naïve; 72 h: 15527±12985% of naïve) and to a lower degree in the contralateral hemisphere (15 min: 249±108% of naïve; 24 h: 2247±1943% of naïve; 72 h: 689±281% of naïve).

In contrast to the latter genes, IL6 expression was regulated differently (**Figure 4 E, F**). 15 minutes after TBI expression was not changed ipsilateral (2 months: 228±54% of naïve; 21 months: 251±70% of naïve) or contralateral (2 months: 210±78% of naïve; 21 months: 155±64% of naïve) to the lesion. In the damaged hemisphere IL6 expression increased independent of age (2 months: 15147±14930% of naïve; 21 months: 10018±9360% of naïve) and remained elevated after 72 hours (2 months: 3985±1575% of naïve; 21 months: 4241±3144% of naïve). In the contralateral hemisphere there was no significant increase at 24 hours after CCI (2 months: 592±570% of naïve; 21 months: 689±623% of naïve). Young animals showed an increase at 72 hours after TBI (3942±2074% of naïve), whereas in old animals contralateral IL6 gene expression returned to baseline levels 72 hours after CCI (177±134% of naïve). Thus, there is an early and persistent up-regulation of IL6 in both age groups in the ipsilateral hemisphere, but only a late increase in young animals after 72 hours in the contralateral region.

### Intracerebral T cell migration

**Figure 5:**
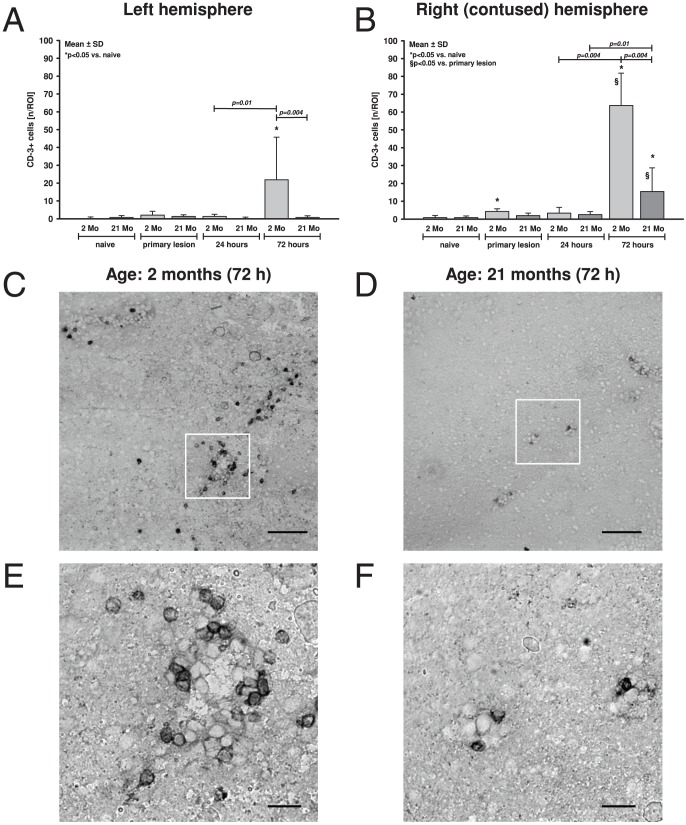
Intracerebral T cell migration in young and old animals after TBI. T cells (CD3^+^ cells) were immunostained and counted (absolute number, n) in coronal slices of brain tissue in young (2 months, light grey) and old (21 months, dark grey) C57/Bl6 mice, in animals without intervention (naïve) and at 15 minutes ( = 15 m, n = 6), 24 hours ( = 24 h, n = 7), and 72 hours ( = 72 h, n = 7) after CCI. Sections were analyzed at bregma level −1.28 mm using a 10× object lens and by counting the number of positive cells in the view field in pericontusional region of interest (ROI) adjacent to the damaged brain tissue and in corresponding contralateral tissue. Cell count in naïve mice was not different between age groups and hemispheres. In the left (contralateral) hemisphere CD3^+^ cell number was similar to naïve levels within the first 24 hours after TBI in both age groups. After 72 hours there was a significant increase of CD3^+^ cells on the contralateral hemisphere of young mice, whereas in old animals CD3^+^ cells remained at baseline levels (**A**). In the right (contused) hemisphere a significant higher CD3^+^ T cell migration was detected very early in young mice and reached a maximum after 72 hours. In old animals in the ipsilateral hemisphere T cell migration did not reach significance until 72 hours after TBI. However, level of pericontusional CD3^+^ cells were higher in young, than old animals (**B**; p<0.05; p-values were adjusted for multiple comparison by Holms-Bonferroni). Typical pictures in two scales (**C** and **D**: scalebar 100 µm; **E** and **F**: scalebar 20 µm) of brain slices with CD3^+^ labeled cells in 2 months (**C**, **E**) and 21 months old mice (**D**, **F**) demonstrate a marked increase of CD3^+^ cells in young animals in contrast to old mice, 72 hours after TBI.

T lymphocytes were immunostained (CD3-positive cells) in coronal slices of brain tissue of both age groups (naïve, 15 minutes, 24, and 72 hours after experimental TBI). Sections were analyzed at bregma level −1.28 mm using a 10× object lens and by counting the absolute number (n) of CD3-positive (CD3^+^) cells in the pericontusional and contralateral region (**Figure 5 C–F**). Average baseline (naïve) cell count was not significantly different between age groups in right (2 months: 0.8±1.3 n/ROI; 21 months: 0.8±1.0 n/ROI) and left hemisphere (2 months: 0.3±0.6 n/ROI; 21 months: 0.8±1.0 n/ROI). In the pericontusional tissue a significant CD3^+^ T cell migration could be detected very early in young mice, at 15 minutes after TBI (4.3±1.5 n/ROI; p<0.05). At 24 hours CD3^+^ cell number sustained (3.3±3.2 n/ROI) and reached a maximum after 72 hours (63.7±18.1 n/ROI; p<0.05 vs. naïve and 15 minutes). In old animals T-cell migration into the traumatized hemisphere did not reach level of significance until 72 hours after TBI (15 min: 1.9±1.5 n/ROI; 24 h: 2.4±1.7 n/ROI; 72 h: 15.4±13.3 n/ROI). However, the number of pericontusional CD3^+^ cells was higher in young compared to old animals (p = 0.05). In the contralateral hemisphere CD3^+^ cells remained at naïve level within the first 24 hours after TBI (15 minutes–2 months: 2.0±2.2 n/ROI vs. 21 months: 1.3±0.9 n/ROI; 24 hours–2 months: 1.2±1.3 n/ROI vs. 21 months: 0.4±0.5 n/ROI). After 72 hours there was a significant increase of CD3^+^ cells in the contralateral hemisphere of young mice, whereas in old animals CD3^+^ cells remained at baseline level (2 months: 21.9±23.9 vs. 21 months: 0.8±0.9 n/ROI; p<0.05, **Figure 5**).

### Pulmonary mRNA expression of IL1β, IL6, COX2 and iNOS

IL1β expression in the lungs was up-regulated in young animals (15 min: 187±69% of naïve; 24 h: 265±140% of naïve; 72 h: 318±145% of naïve; p<0.05 vs. naïve), whereas expression was not up-regulated in old animals (15 min: 58±17% of naïve; 24 h: 123±67% of naïve; 72 h: 119±71% of naïve). In lungs gene expression of IL6 was not altered at any time point in young (15 min: 185±22% of naïve; 24 h: 92±72% of naïve; 72 h: 155±111% of naïve) and old mice (15 min: 143±41% of naïve; 24 h: 125±120% of naïve; 72 h: 107±110% of naïve). Similarly, pulmonary COX-2 expression was not influenced by cerebral injury in young (15 min: 95±26% of naïve; 24 h: 122±45% of naïve; 72 h: 119±37% of naïve) and aged animals (15 min: 103±43% of naïve; 24 h: 103±46% of naïve; 72 h: 93±29% of naïve). Pulmonary expression of iNOS decreased at 24 and 72 hours after brain injury in young animals (15 min: 124±26% of naïve; 24 h: *91±20% of naïve; 72 h: *82±25% of naïve; *p<0.05 vs. primary injury) and remained unchanged in old mice (15 min: 121±42% of naïve; 24 h: 142±69% of naïve; 72 h: 124±33% of naïve).

### Hematologic assessment

The results of the hematological assessment are depicted in **Table 4**. Hemoglobin levels were similar in both age groups without significant differences. Platelet numbers remained unchanged throughout the experimental period in old and young mice.

**Table 4:** Quantification of blood differential cell count.

	2 mo	21 mo	2 mo	21 mo	2 mo	21 mo	2 mo	21 mo
	naïve	naïve	15 min	15 min	24 h	24 h	72 h	72 h
**Hemoglobin**	12.2±0.6	11.4±1.0	13.2±0.4	11.3±1.0	12.5±0.8	10.9±1.4	12.5±0.5	10.7±2.4
**[g/dl]**								
**Platelets**	979±244	1617±447	1250±150	1485±589	1026±281	1226±753	1013±369	1269±381
**[10^3^/µl]**								
**White Blood**	3.8±1.9	3.5±2.0	6.6±1.7	**4.7±1.9^§^**	4.1±1.2	**2.6±1.3^§^**	3.7±1.4	5.0±2.6
**Cells**								
**[10^3^/µl]**								
**Lymphocytes**	87.3±5.8	60.0±12.1	70.1±6.1	57.9±20.6	78.3±7.5*	35.4±15.8*	69.8±8.6	48.7±29.2
**[%]**								
**Neutrophil**	4.3±2.0	28.4±11.8	3.3±2.7	13.5±11.4	7.2±4.9*	56.0±15.8*	8.3±6.0	14.1±9.7
**Granulocytes**								
**[%]**								
**Basophil**	0.48±0.3	0.68±0.5	0.95±0.1	1.05±0.3	0.73±0.4	0.54±0.2	0.77±0.4	0.72±0.6
**Granulocytes**								
**[%]**								
**Eosinophil**	1.92±1.7	0.90±1.0	0.50±0.6	1.18±1.3	1.35±1.5	3.06±2.3	1.63±1.7	0.16±0.1
**Granulocytes**								
**[%]**								
**Monocytes**	3.37±3.5	6.55±7.0	11.25±3.2	18.37±24.7	12.50±15.7	1.19±0.8	12.40±12.4	33.94±17.5
**[%]**								

Hemoglobin concentration and platelets were not significantly different at any time point between age groups (young: 2 months = 2 mo, old: 21 months = 21 mo). Number of white blood cells (WBC) was by trend higher 15 minutes after TBI. In old animals 24 hours after TBI WBC were reduced indicating a systemic CNS injury-induced immunodepression (CIDS; **^§^**p<0.05 in old animals (21 months): 15 min vs. 24 h). In young animals reduction of WBC did not reach level of significance (p = 0.067). Lymphocytes were significantly lower whereas neutrophil granulocytes levels were relatively increased in old animals compared to young animals after 24 hours (*p<0.05, 24 h: 2 mo vs. 21 mo). Basophil, eosinophil granulocytes and monocytes were not regulated differently in old and young mice at any time point (Data are presented as mean±SD).

In both age groups white blood cell count (WBC) increased 15 minutes after TBI without reaching level of significance (p = 0.067 and p = 0.381 in young and old mice, respectively). In old animals 24 hours after TBI, WBC were down-regulated compared to 15 minute values (p<0.05). In young animals the reduction of WBC failed to reach level of significance (p = 0.067).

Lymphocytes were significantly down-regulated whereas neutrophil granulocytes were relatively up-regulated in old animals compared to young animals after 24 hours (*p<0.05, 24 h: 2 months vs. 21 months). Basophil, eosinophil granulocytes and monocytes were not regulated differently in old and young mice at any time point, although there was a trend towards down-regulation of monocytes at 24 hours in old animals.

## Discussion

One major shortcoming in the field of TBI research is the lack of experimental studies that utilize rodents beyond the age of 3–6 months. Results of clinical studies on human TBI suggest the need for increased research efforts on aged individuals [16]–[18]. The few experimental studies on TBI indicate that age at the time of injury is a major determinant for increased injury severity and attenuation of the subsequent recovery [13], [19]–[25]. However, the underlying pathological mechanisms behind these observations are still poorly understood [9].

The aim of the present study was to clearly determine the influence of age on posttraumatic brain lesion expansion, edema formation, neurological function, and inflammatory markers in the brain, in lung as a remote organ, and in the blood. The aged animals (21 months) that were used in the present investigation originated from the same breed as the young adult mice. The Kaplan Meyer curve of the aging animals shows (**Figure 1**) a strong increase of the natural mortality rate during the last months before start of the experiments. The high mortality rate was therefore the limiting factor for the number of animals available for this study. To induce TBI in mice, various focal lesion models have been developed, *e.g.* freezing or fluid percussion injury [26]–[28]. For the present study the CCI model was selected due to its very high reproducibility and histopathological similarity to contusion injuries in human TBI patients [27], [29]. Cerebral lesion expands into surrounding healthy parenchyma within 24 hours after insult [30], [31]. Therefore, 24 and 72 hours were chosen as observation time points to include also a late time point to determine the final brain lesion size and neurological dysfunction.

Long-term cognitive recovery and motor dysfunctions have been recognized as the most debilitating consequences of human TBI [32]. Similar to clinical data which show a poorer functional outcome in aged patients [5], [6], [16], [32], [33] and data showing an exacerbation of cognitive and functional decline following TBI in both aged humans and rodents [34]–[36], the present study reveals a higher mortality rate and worse neurological outcome in aged animals. These findings are corroborated by differences in change of body weight between both age groups and are in line with other studies investigating experimental TBI and cerebral ischemia [9], [13], [37]. However, even before TBI old animals showed an impaired motor function. This has already been described by Barreto and coworkers [38] who characterized locomotor activity and spatial learning deficits in healthy aged mice.

However, poorer neurological outcome did not correlate with histopathological damage; contusion volumes were equal in both age groups at all time points after TBI. This in accordance with the results of a CCI study performed by Onyszchuk and coworkers, where lesion cavity size was similar in both age groups (6 and 24 months) at 3 days after injury [13], indicating that acute loss of brain tissue is not the cause of greater functional deficits in aged traumatized animals. In the present study a more severe brain edema formation was detected in old mice at 24 hours after TBI, which also failed to correlate with neurological deficits. This indicates that brain edema formation is not primarily responsible for age dependent differences in neurofunctional outcome.

One possible explanation for the neurological deterioration at 72 hours post insult of aged animals is reduced functional reserve. In contrast to greater body size and weight greater in the aged groups; brains of old naïve mice were about 8% smaller compared to young naïve brains. This suggests an age-related cerebral atrophy with possible decline in cerebral plasticity as an additional cause for the poor functional recovery of old animals. In several experimental TBI studies activation of neurotrophic factor-signaling pathways reduce neuronal damage and improve functional outcome [39]. These findings implicate neuronal plasticity as one general mechanism for recovery in the central nervous system [40]. Neuronal plasticity and neurogenesis can be enhanced in aging rodents by enriched environment [41]. Buchold and coworkers revealed beneficial effects of an enriched environment on infarct size and neurological recovery in young and aged rats, but with reduced efficacy in aged animals [42].

Possible alternative explanations for worse neurological outcome of aged mice after 72 hours post insult may be secondary neuronal dysfunction or differences in posttraumatic inflammatory response on the cerebral and systemic level. Therefore, in addition to cerebral inflammation, white blood cells count (WBC) and pulmonary inflammation was assessed. In old animals 24 hours after TBI total WBC was reduced indicating a systemic CNS injury-induced immunodepression (CIDS). Furthermore, lymphocytes levels were significantly lower whereas number of neutrophil granulocytes was relatively increased in old animals compared to young mice at 24 hours after injury. The mechanisms that underlie these age-related effects have been attributed in the literature to general changes ranging from defects in the hematopoietic bone marrow to defects in peripheral lymphocyte migration, maturation, and function. Chronic involution of the thymus gland is thought to be one of the major contributing factors to loss of immune defense with increasing age [43]. Immunosenescence is a complex process during aging that negatively impacts the immune system and its ability to adapt to age-associated changes. During aging thymus involution leads to a shift of immune reaction from a T cells- and B cell-based immune reaction towards a non-specific cellular reaction by neutrophil granulocytes.

In the pulmonary tissue a different age-dependent immune reaction pattern could be detected. Gene expression of IL1β was increased and iNOS expression down-regulated in young animals, whereas in old animals expression levels were unchanged after CCI. In contrast, expression of IL6 and COX-2 was not altered in the lungs after TBI. The lack of IL1β up-regulation in the lung tissue as well as a reduction in WBC of old animals suggests the presence of a CIDS. After brain injury the disturbance of the interplay between the immune system and the CNS that leads to disruption of signaling in the control circuitry of neural–immune interactions, resulting in secondary immunodeficiency with a down-regulation of innate and adaptive immunity [44]. Clinical and experimental evidence points towards a major role of impaired cell-mediated immune responses in the high incidence of infectious complications after CNS injury [44].

In the present study, mRNA expression levels of IL1β, TNFα and IL6 were determined in both hemispheres as general marker for neuroinflammatory response. Baseline expression levels were already significantly higher in old mice compared to young mice. This is in line with reports on increased basal expression of cytokines and chemokines in aging brains [23], [45], [46], which seems to be related to an increase of constitutive NF-κB DNA binding activity in forebrain and hippocampus of aged rats [21]. The aging brain is characterized by a shift from the homeostatic balance of inflammatory mediators to a pro-inflammatory state [9]. A higher level of inflammatory cytokines in the present study may indicate a subclinical chronic inflammatory process in the elderly, referred to in the literature as “inflamm-aging” [15]. An elevated neuroinflammatory level sets the stage for an exaggerated inflammatory cytokine response after neuronal insult that may lead to behavioral and cognitive deficits [24], [47].

Aging can substantially exaggerate the systemic inflammatory responses, *e.g.* wound healing [48] and the inflammatory reaction of the CNS [22], [23]. Following injury increased inflammatory responses of injured cortical tissue was described in several TBI studies, *e.g.* as result of a stab wound [22]. Following TBI an exaggerated glial activation occurs in aged brain [24]. The profound neuroinflammatory response after TBI may serve to defend the brain from invading pathogens and to promote neural repair. However, it contributes to the development of cerebral edema formation by blood-brain barrier breakdown [13], [49], increased oxidative stress with mitochondrial dysfunction, and decreased endogenous protection against free radical production in aged individuals following brain injury [50]–[53]. After TBI in old animals an altered neurotrophin metabolism and signaling [54] and age-related changes in astrocytes and microglia activity have likewise been implicated to worsen outcome [13], [47], [55] and ultimately leading to neuronal cell death. However, aside from these deleterious effects, neuroinflammation has also been shown as beneficial after brain injury [56]–[58].

In the brain mRNA expression of the cytokines IL1β, TNFα and IL6 increased after CCI in the present study. However, the expression pattern was different in young compared to old mice. In old animals IL1β and TNFα expression increased early and ipsilateral to the damage, whereas in young animals delayed expression appeared after 3 days and in both hemispheres. A similar inflammatory response pattern with elevated IL1β expression [23] and with early up-regulation of CCAAT/enhancer binding proteins (types β and γ) and consecutive early inflammatory response was shown in aged mice [25]. Injection of IL1β at the time of experimental injury caused increased cell death and edema [59] and early increased TNFα levels were associated with neurologic deficits in rodents subjected to experimental brain injury [60]. Both TNFα and IL1β have been shown to promote tissue infiltration of neutrophil granulocytes and induce endothelial cell adhesion molecule expression and to activate pathways upstream to neuronal apoptosis [61], [62]. Early and high expression of these cytokines in aging mice could synergistically induce apoptosis and lead to increased secondary neuronal cell death. Increased inflammatory response in aging brain may differ upon mechanical injury from the natural age-related changes or in response to pure inflammatory stimuli. The present study demonstrates an increased inflammatory response during increase of secondary brain injury. This is in contrast to *e.g.* the inflammatory response to LPS injections, which is reduced in aged mice [46]. In experimental brain injury, old animals showed an earlier, exacerbated astroglial response at day 1 post-injury, a larger area of astrogliosis and greater glial scar compared to young animals [63]. Both astrocytes and macrophages are activated strongly and early following stroke in aged rodents [9]. In older animals, the premature, intense cytoproliferative activity following brain injury leads to the precipitous formation of growth-inhibiting scar tissue, a phenomenon amplified by the persistent expression of neurotoxic factors.

TNFα is a pleiotropic cytokine involved in inflammatory cascades at different phases after CNS injury. Growing evidence suggests that early neuronal expression of TNFα after TBI contributes to subsequent neurological dysfunction [60], whereas later expression of TNFα is linked to improved outcome [57], [64]–[67]. These opposing effects of TNFα may be partly explained by the existence of two distinct signaling pathways mediated through TNFα receptor 1 (TNFR1) and a potentially beneficial TNFR2 [66], [68].

In the present study IL6 was regulated differently and demonstrated an early up-regulation in the pericontusional cortex in both age groups, which sustained after 3 days. Increasing evidence supports a key role of IL6 in neuronal survival, differentiation, regeneration and degeneration in the peripheral and central nervous system [58]. Prolonged expression of IL6 after brain injury may reflect ongoing inflammation or could be a beneficial mediator of anti-inflammatory response. It is known that cytokines are involved in nerve regeneration by modulating the synthesis of neurotrophic factors [57]. IL6 seems to have anti-inflammatory effects, and its neurotrophic properties are supported by its ability to inhibit TNF synthesis, induce nerve growth factors, promote neuronal differentiation and survival, and counteract N-methyl-D-aspartate–mediated toxicity [69]. In addition, the constitutive expression of IL6 and of its receptor in the healthy brain indicates a possible role in maintaining a normal physiologic function of the nervous system independent of neuropathology [57].

The present data reveals an age-dependent pattern of T cell migration. Cluster of differentiation 3 (CD3) is part of the T cell receptor complex and a highly specific immunohistochemical marker for mature T cells in tissue sections. In young animals an early increase of CD3^+^ cells could be detected in the pericontusional tissue. After 72 hours there was a second increase to higher levels compared to 24 hours. In the contralateral hemisphere there was a less pronounced increase after 72 hours. In contrast to young mice, old animals showed a lower increase of T cells in the pericontusional tissue after 3 days whereas in the contralateral hemisphere T cell migration could not be observed. Therefore, similar to the cytokine expression pattern, quantification of CD3-positive T cells reveals a bilateral neuroinflammatory response with respect to T cell migration on the third posttraumatic day in young animals compared to a more unilateral posttraumatic inflammatory response in aged animals.

T cells play a vital role in mediating cellular and humoral immunity. In aged individuals immunosenescence leads to a change of immune reactions on cellular level. Interactions between lymphocytes and antigen presenting macrophages and microglia are disturbed. Older rats show a delayed recruitment of phagocytic cells and diminished clearance of myelin. These deficits correlate with slower remyelination in older animals. Therefore, it is conceivable that changed inflammatory reaction in aged rodents in response to brain injury impedes the removal of cellular debris, thereby hindering tissue restoration, particularly axonal growth and remyelination [9]. An important cellular event associated with reduced structural and functional recovery after stroke in aged animals is an accelerated inflammatory reaction and an early scar formation that impairs subsequent neural recovery and repair [9], [10], [42], [47]. Furthermore, recent findings indicate that immune cells are able to produce a variety of neurotrophic factors, which promote neuronal survival and may also mediate anti-inflammatory effects. [66]. In several studies neuroprotective effects for T cells after brain injury were suggested [66], [67]. Activated T cells have been shown to reduce the spread of damage. This neuroprotective effect of T cells may be caused, at least in part, by the production of neurotrophic factors such as neurotrophin-3 or brain-derived neurotrophic factor. As Bieber and coworkers demonstrated, T cells are necessary for efficient remyelination [67]. In the light of these findings, it is conceivable, that lack of marked T cell migration contributed to worse outcome in old animals. Moreover, recent studies demonstrated that neuroprotection was not observed in the absence of mature T cells [66].

## Conclusions

The present study suggests that beyond histopathologically detectable secondary brain damage, other mechanisms may be responsible for impaired neurological outcome of aged mice following TBI. Reduced plasticity of old brains, a higher degree of edema formation, and a changed pattern of inflammatory response with differences in T cell migration and pleiotropic inflammatory cytokines expression and an earlier onset and longer lasting cerebral inflammatory response may contribute to the poor functional recovery of old animals [9], [13], [23], [24], [37]. The ability of aged brains to compensate neuronal cell loss after brain trauma is largely impaired. Therefore, the effects of age on recovery from TBI need to be addressed [70] to develop therapeutic interventions that aim on the specific problems of old brains. Strategies to understand and reduce age-specific differences may help to develop pharmacologic intervention for the aged population.

## Materials and Methods

A total of 73 male C57Bl6N mice (Charles River Laboratory, Sulzfeld, Germany) were investigated. Young adult mice (2 months old; 21.2±2.0 g) were compared to aged animals, 21 months of age (46.8±7.8 g). This study was performed in accordance with institutional guidelines of the Johannes Gutenberg-University, Mainz. All efforts were made to minimize suffering and the number of animals. The Animal Care and Ethics Committee of the Landesuntersuchungsamt Rheinland-Pfalz approved all experiments (protocol number: G07-1-021). Animals were kept under controlled light and environmental conditions (12 h dark/light cycle, 23±1°C, 55±5% relative humidity), and had access to food (Altromin, Germany) and water ad libitum during all times before and after the experiments.

### Traumatic brain injury and experimental groups

Animals were anesthetized using sevoflurane (4 vol%, i.e. 2 MAC) in an air/oxygen mixture (40% O_2_ and 60% N_2_), which was supplied via facemask in spontaneously breathing animals. Rectal temperature was maintained constant at 37°C by a feedback controlled heating pad (Hugo Sachs, March-Hugstetten, Germany). Experimental brain trauma was induced with a controlled cortical impact (CCI) device as previously described [30], [71]–[73]. Animals were fixed in a stereotactic frame (Kopf Instruments, Tujunga, USA) and a large craniotomy (4×4 mm) was drilled using a saline-cooled high-speed drill above the right parietal cortex between the sagittal, lambdoid, and coronal sutures and the insertion of the temporal muscle. A custom fabricated pneumatic controlled cortical impactor (L. Kopacz, Mainz, Germany) was placed directly and perpendicular to the brain surface. For all animals an impactor tip with a diameter of 3 mm, an impact velocity of 8 m/s, impact duration of 150 ms and brain penetration depth of 1 mm was used. Immediately after CCI the craniotomy was closed with the initially removed bone and fixed with conventional tissue glue (Histoacryl, Braun-Melsungen, Melsungen, Germany). The wounds were closed with filament sutures. Blood pressure was measured in anesthetized animals 5 minutes before and 5 minutes after CCI and in trained awake animals before CCI, 6 hours and 24 hours after CCI at the tail using a modified NIBP system (RTBP 2000, Kent, USA) as previously described [74]. Cuff pressure signals were recorded with a sample rate of 100 Hz (A/D converter: PCI 9112, Adlink Technology, Taiwan; PC software: Dasylab 5.0, measX, Germany) and analyzed offline (Flexpro 6.0, Weisang, Germany) for systolic blood pressure.

After closure of the wounds, animals of both age groups were randomly assigned to: 15 minutes, 24 and 72 hours survival after CCI and sevoflurane was discontinued. Animals that survived 15 minutes post-CCI remained on the heating pad until their brains were removed and blood had been withdrawn for arterial blood gas analysis for hemoglobin concentration, hematocrit, electrolyte, and blood glucose levels (blood gas analyzer ABL800 BASIC, Radiometer Medical ApS, Brønshøj, Denmark). In the 24 and 72 hours survival groups animals were placed in their individual cage and were allowed to recover for 6 hours in an incubator heated to 33°C and at a humidity of 35% (IC8000, Draeger, Germany). Brain edema formation was assessed 24 hours after experimental TBI. Histological brain damage was determined 15 minutes, 24 and 72 hours after trauma. CCI groups were compared to naïve – non-operated – animals of both age groups. Neurological assessment was performed before trauma and 24 and 72 hours after TBI. Quantification of neuronal inflammation was performed in pericontusional and contralateral brain tissue by gene expression analysis. The following experimental groups were used.

### Experimental groups (target parameters, group size):

1. Naïve animals, no surgery (hematology, neurology, histology, and inflammation):
  2 months: n = 6 (n = 6 survived)
  21 months: n = 6 (n = 6 survived)
2. 15 minutes survival (physiology, hematology, and histology):
  2 months: n = 6 (n = 6 survived)
  21 months: n = 6 (n = 6 survived)
3. 24 hours survival (hematology, neurology, histology, and inflammation):
  2 months: n = 7 (n = 7 survived)
  21 months: n = 7 (n = 7 survived)
4. 24 hours survival (blood pressure, neurology, and brain water content):
  2 months: n = 8 (n = 8 survived)
  21 months: n = 10 (n = 8 survived)
5. 72 hours survival (hematology, neurology, histology, and inflammation):
  2 months: n = 7 (n = 7 survived)
  21 months: n = 9 (n = 7 survived)

### Quantification of functional outcome

Before and after CCI functional outcome was determined by an investigator blinded towards experimental groups by measurement of body weight and assessment of neuroscore. A 10-point neurological severity score (NSS) was applied as described by Tsenter et al. [14]. The neuroscore consists of 10 different tasks evaluating motor ability, alertness, balancing, and general behavior. One point was awarded for failure to successfully perform a task. Healthy mice were successful in all tasks and received 0 points. Severely impaired animals received up to 10 points (**Table 5**).

**Table 5:** Criteria of the Neurological Severity Score (NSS) after Tsenter et al. (2008).

Task	Points
Presence of mono- or hemiparesis	1
Inability to walk on a 3-cm-wide beam	1
Inability to walk on a 2-cm-wide beam	1
Inability to walk on a 1-cm-wide beam	1
Inability to balance on a 1-cm-wide beam	1
Inability to balance on a round stick (0.5 cm diameter)	1
Failure to exit a 30-cm-diameter circle (for 2 min)	1
Inability to walk straight line	1
Loss of startle behavior	1
Loss of seeking behavior	1
Maximum total	10

One point is awarded for failure to perform a task.

### Histological evaluation and immunohistochemistry

At the end of the observation period animals were euthanized in deep anesthesia and brains were carefully removed, frozen in powdered dry ice, and stored at −20°C. Brains were cut in coronal plane with a cryostat (HM 560 Cryo-Star, Thermo Fisher Scientific, Walldorf, Germany). 10 µm thick sections were collected every 750 µm, placed on Superfrost plus slides (Thermo Fisher Scientific, Germany) and stained with cresyl violet. The area of both hemispheres and contused brain tissue were determined with a computerized image system (Optimas 6.51, Optimas Corporation, Bothell, WA) by an investigator blinded towards to experimental groups and contusion volume was calculated by multiplying contusion areas with the distance between histological sections (750 µm). For immunohistochemistry 10 µm coronal cryostat sections were fixed in 4% paraformaldehyde for 10 min, rinsed in PBS, blocked for 1 hour in PBS/5% goat serum/5% horse serum/0.5% BSA/0.1% Triton X-100 and incubated overnight with antibodies specific to CD3 (rat anti-CD3, Zytomed, Germany, diluted 1∶500 in blocking solution). Sections were washed, incubated with secondary biotin-conjugated antibodies and processed according to the instructions of the manufacturer using Vectastain Elite ABC Kit (Vector laboratories, Inc. Burlingame, CA). The total amount of CD3-positive cells were counted in the cortical pericontusional and corresponding contralateral tissue at bregma – 1.28 mm according to the Mouse Brain Library (http://www.mbl.org) by two investigators blinded to the group allocation. Therefore a region of interest (ROI) was chosen at the border zone adjacent to the damaged brain tissue and in the corresponding area in the left (contralateral) hemisphere (ROI: 0.5×0.5 mm).

### Quantification of Brain Water Content

Animals were reanesthetized 24 hours after CCI. After removal of the brains, cerebellum was separated and hemispheres were cut along the interhemispheric plane. Both hemispheres were weighed to assess their wet weight. The hemispheres were dried for 24 hours at 110°C to determine the dry weight. On the basis of the gravimetrical differences, brain water content was obtained by the following calculation [75]: Hemispheric water content (%): (WW - DW)/WW ×100, where WW is the wet weight (g) and DW, the dry weight (g) of the brain hemispheres.

### Hematologic Assessment

Blood samples were taken from the left heart ventricle under deep anesthesia before decapitation as described above and stored in EDTA. Blood cells were counted automatically by fluorescence activated cell sorting (FACS). Hematologic assessment was performed in the laboratory of the Institution for Laboratory Medicine of the Medical Center of the Johannes Gutenberg University Mainz with especially programmed FACS devices for rodent blood samples.

### Gene expression analysis

For the determination of gene regulation (mRNA quantification), brain and lung tissue samples were taken during histological cryosectioning (−20°C). Samples from both hemispheres were separately collected, frozen in liquid nitrogen, and stored at −80°C. Brain tissue was lysed in RNeasy lysis buffer (Qiagen, Hilden, Germany) and homogenized with a MM300 mill mixer (Retsch, Haan, Germany). Total RNA was isolated from the lysed and homogenized cells using RNeasy Lipid Tissue Mini Kit (Qiagen) and eluted with 25 µL of RNase-free water. RNA concentration was spectrometrically calculated using NanoVue (GE Healthcare Europe, Munich, Germany). Thereafter, 0.5 µg extracted RNA was reverse-transcribed into cDNA by Verso™ cDNA Kit (ABgene, Hamburg, Germany). PCR fragments of all applied genes were generated by PCR on a Thermocycler gradient (Eppendorf, Hamburg, Germany). To verify the specificity of the PCR reaction, PCR products were electrophoresed alongside the 50 bp DNA Molecular Weight Marker XIII (Roche Diagnostics, Mannheim, Germany) through a 2% (w/v) agarose gel (Invitrogen). The gels were stained with SYBR green (Roche) and images were captured using a Kodak EDAS 120 Image System (Eastman Kodak Sàrl, Genève, Switzerland). The PCR products were purified with QIA quick PCR Purification Kit (Qiagen) and the DNA concentration was determined using a NanoVue system (GE Healthcare). The copy number was calculated and serial 10-fold dilutions were made in the range of 1×10^7^ to 1×10^1^ copies. cDNA of each sample was amplified by a real-time Lightcycler 480 PCR System (Roche). Equal amounts of cDNA (1 µl) were used in duplicates and amplified with Lightcycler 480 Probes Master (Roche). Applied primers and probes are listed in **Table 6**. A standard curve for absolute quantification was generated with PCR DNA for each PCR product. The absolute copy numbers of the target genes was normalized against the absolute copy numbers of Cyclophilin A (PPIA) as control gene [72].

**Table 6:** Specific primer and probes and optimized temperature conditions for real-time PCR.

PCR assay	Oligonucleotide Sequence (5′-3′)	GenBank
(amplicon size, annealing temp, A, E)		No. (Ref)
Cyclophilin A (PPIA)	Forw: 5′-GCGTCTSCTTCGAGCTGTT-3′	NM_008907
	Rev: 5′-RAAGTCACCACCCTGGCA-3′	
(146 bp, 55°C, A: 10 s, E: 15 s)	Cy5: Red-TTGGCTATAAGGGTTCCTCCTTTCACAG-Phos	[72]
	FL: 5′-GCTCTGAGCACTGGRGAGAAAGGA-Fl	
TNFα	Forw: 5′-TCTCATCAGTTCTATGGCCC-3′	NM_013693
(212 bp, 62°C, A: 10 s, E: 10 s)	Rev: 5′-GGGAGTAGACAAGGTACAAC-3′	[76]
IL6	Forw: 5′-GAGGATACCACTCCCAACAGACC-3′	NM_031168
(141 bp, 58°C, A: 10 s, E: 10 s)	Rev: 5′-AAGTGCATCATCGTTGTTCATACA-3′	[77]
IL1β	Forw: 5′-gTgCTgTCggACCCATATgAg-3	NM_008361
	Rev: 5′-CAggAAgACAggCTTgTgCTC-3′	
(348 bp, 55°C, A: 10 s, E: 15 s)	Cy5: Red-CAgCTggAgAgTgTggATCCCAAgC-Phos	[78]
	FL: 5′-TAATgAAAgACggCACACCCACCC-Fl	
iNOS (NOS2)	Forw: 5′-TGTGTCAGCCCTCAGAGTAC-3′	NM_010927
	Rev: 5′-CACTgACACTYCgCACAA-3′	
(312 bp, 58°C, A: 10 s, E: 15 s)	Red: R610-gCTCCTCCCAggACCACACCC-Phos	[79]
	FL: 5′-gAAgCCCCgCTACTACTCCATC-Fl	
Cyclooxygenase 2 (PTGS2)	Forw: 5′-TCTTTgCCCAgCACTTC-3′	NM_011198
	Rev: 5′-CCTCTCCACCRATgACCTgA-3′	
(183 bp, 55°C, A: 10 s, E: 15 s)	Red: R610-ggTCCTCgCTTMTgATCTgTCTTgAA-Phos	[79]
	FL: 5′-CCAgTCCTCgggTgAACCC-Fl	

### Statistical Analysis

Contusion volumes, brain water content, CD3+ cell count and mRNA expression data were compared between experimental groups with Wilcoxon Mann Whitney Rank Sum Tests and p-values were adjusted for each parameter for multiple comparisons (Bonferroni adjustment) by multiplication of the test results (p values) with the number of performed tests (brain water content, lesion volume and neurological function score: 5 tests each; mRNA expression data: 5 tests, with respect to the number of tests (n = 16), in CD3^+^ cell count a Bonferroni-Holms correction was performed). Physiologic data were analyzed with one-way analysis of variance. To investigate the correlation between brain water content and neurological function a nonlinear polynomial regression analysis was performed. Results are presented as mean ± standard deviation (SD). Differences were considered significant at p<0.05. The study was conducted in an explorative approach; all p values are therefore considered as explorative. Statistical analysis was performed with Sigma Plot 11 Statistical Software package (Systat Software Inc., San Jose, CA).
